# The relationship between body mass index, body image, and sexual function: A survey on Iranian pregnant women

**DOI:** 10.18502/ijrm.v17i7.4862

**Published:** 2019-07-31

**Authors:** Mahtab Senobari, Elham Azmoude, Marziyeh Mousavi

**Affiliations:** ^1^Student Research Committee, Torbat Heydariyeh University of Medical Sciences, Torbat Heydariyeh, Iran.; ^2^Department of Midwifery, School of Nursing and Midwifery, Torbat Heydariyeh University of Medical Sciences, Torbat Heydariyeh, Iran.; ^3^Health Sciences Research Center, Torbat Heydariyeh University of Medical Sciences, Torbat Heydariyeh, Iran.

**Keywords:** Body mass index, Body image, Sexual function, Pregnancy.

## Abstract

**Background:**

The prevalence of sexual problems is high during pregnancy. Despite this, there are limited data about the impact of physical and psychological factors such as body weight and body image on sexual function in pregnant women.

**Objective:**

To investigate the relationship between body mass index, body image, and sexual function among pregnant women.

**Materials and Methods:**

In this cross-sectional study, a total of 206 Iranian pregnant women (106 with normal weight and 100 overweight women) in their 2nd and 3rd trimesters of pregnancy were surveyed. Survey instruments included the Female Sexual Function Index and Multidimensional Body-Self Relations Questionnaire.

**Results:**

The prevalence of female sexual disorder was 72.3% in this survey. Diminished sexual desire/appetite was the most common problem reported by the participants (37.9%). The mean score of sexual problem and body image were not significantly different among overweight and normal weight women in the 2nd (p = 0.945 and p = 0.800, respectively) and 3rd trimesters of pregnancy (p = 0.310 and p = 0.507, respectively). Further, there were no relationships between the body mass index plus body image and the total female sexual function score (p = 0.44 and p = 0.837, respectively). However, the relationship between the appearance evaluation with lubrication (p = 0.043) and subjective weight with two subscales of sexual satisfaction (p = 0.005) and orgasm (p = 0.019) were significant.

**Conclusion:**

The findings from this study revealed that there were no relationships between body mass index plus body image score and the sexual function in pregnancy. Therefore, a further research is recommended to study other potential factors affecting sexual function during pregnancy.

## 1. Introduction

Pregnancy is an important life event associated with profound physiognomic and psychosocial changes that might influence the sexual intimacy in expectant mothers (1). Some pregnant women have a wrong impression that sexual relationships during pregnancy would hurt the mother and the fetus (2). In addition, many women report a decline in sexual frequency and impaired sexual function during pregnancy and even several months after childbirth (3). In a study, it was found that 79.1% of the Iranian pregnant women had sexual problem (4). This is while only sparse evidence has been published about the potential harms of sexual intercourse during pregnancy (5). Generally, the sexual function impairment during pregnancy or at any time point may lead to various detrimental consequences, including conflict and tensions in the couple's relationship, family disputes, and ultimately separation and divorce (6). Unfortunately, despite the widespread prevalence and consequences of this problem, sexual function is not always addressed during a routine prenatal care (7).

Sexuality, as a complex phenomenon, may be affected by a number of factors. Extensive literature is available on these factors in non-pregnant women (8, 9). However, these associations are less clear in pregnancy. Cultural norms, religious beliefs, myths and fear, and perception of the body may affect the sexuality of pregnant women (10, 11). For instance, there are several lines of evidence suggesting that greater body satisfaction is associated with higher perceived sexual desirability, more frequent sexual experiences, more interest in engaging in sexual activity, and fewer sexual problems (10). Pregnancy creates fundamental changes in a woman's body size and shape (12). Accordingly, body image disturbance has been shown to be common in this stage (13). Research suggests that body dissatisfaction is associated with the symptoms of depression and anxiety, low self-esteem, and eating disorders (14).

One of the other serious public health problems which has the potential to promote sexual problem in reproductive age women is obesity (8). As with many other developing countries, Iran is facing the epidemics of obesity and its complications. Specifically, the prevalence of obesity is estimated to be 21.7% in Iranian adult populations (15). During pregnancy, excessive maternal weight may adversely affect pregnancy outcomes (16). Obese pregnant women have risk factors which may increase the risk of development of sexual problem symptoms (1, 17). In another study, it was shown that overweight women had a weaker sexual function score than women with the normal weight in the third trimester (17). Meanwhile, to the best of our knowledge, limited data and resources exist on the effects of obesity on the sexual function of pregnant women. Further, the existing literature has mostly been conducted in Western environment and there are no available studies on this topic in Iran (1, 17).

According to the aforementioned points, it is important to understand how the perception of the body and body weight affect the sexuality of women during pregnancy to improve the sexual well-being of couples by providing guidance and appropriate care during this period of life. The aim of the current study was to investigate the relationship between body mass index (BMI), body image, and sexual function among pregnant women.

## 2. Materials and Methods

This cross-sectional study was conducted in healthcare centers in Iran between August and November 2017. A total of 206 pregnant women participated in this study using a non-probability convenience sampling. The sample size was determined using a pilot study based on the level of significance of (α) = 5%, β = 20% (power = 80%), and sample size formula in correlation studies. The eligibility criteria of the study were: age 20 yr or older, singleton pregnancy, gestational age 14 wk or more (based on early ultrasound before 20 wk gestation or last normal menstrual period), and pre-pregnancy BMI ≥ 18.5 kg/m${}^{2}$ (based on the recorded pre-pregnancy weight and measured height).

Women with chronic diseases such as diabetes, hypertension, and heart disease, as well as those with pregnancy-related complications such as preeclampsia were excluded, alongside women with a current or history of psychiatric disorder, substance abuse, admission to the hospital in the previous month, and those in sexual abstinence due to a medical reason or absence of husband. The research data were collected using three anonymous, written questionnaires. The demographics form included questions such as age, spouse's age, educational level, occupation status, and obstetric characteristics such as the number of pregnancies, history of miscarriage, gestational age, and pre-pregnancy BMI.

The Female Sexual Function Index (FSFI) as well as Body Image Scale was also completed. The FSFI is a validated self-reported questionnaire with 19 questions employed to assess the participant's sexual function over the past four weeks. Each question is scored ranging from a minimum of 0 or 1 to a maximum of 5. This scales included six domains of desire, arousal, lubrication, orgasm, satisfaction, and sexual pain. The full scale score varied from 2 to 36 and was calculated by summation of the six subscale scores. Higher scores indicated a better sexual function (18). The validity and internal reliability tests for the Iranian version of the FSFI had been undertaken by Mohammadi and colleagues, where the Cronbach's alpha coefficient was found to be 0.87 (19). The Multidimensional Body-Self Relations Questionnaire (MBSRQ) is a 46 item scale, which assess body image. Each item scored on a 5-point Likert scale ranging from 1 (strongly disagree) to 5 (strongly agree). The score of the questionnaire was between 46 and 230, with higher scores demonstrating a higher body satisfaction. This included six subscales of appearance evaluation, appearance orientation, fitness evaluation, fitness orientation, subjective weight, and body areas satisfaction (20). The validity and reliability of this scale had already been confirmed by Rahati and colleagues across Iranian population in 2004 (21). In the present study, the internal consistency of this scale and all subscales were established during the pilot study with a Cronbach's ≈ ranging 0.76-0.97. Gestational age was estimated base on the last normal menstrual period or early obstetric ultrasound before 20 wk gestation. The heights of the participants were measured without shoes. BMI was computed based on weight in kilograms divided by square of the height in meters. The pregnant women were categorized into two groups: normal weight and overweight/obese.

### Ethical considerations

This study was approved by the ethical committee of Torbat Heydariyeh University of Medical Sciences (Code: IR.THUMS.REC.1395.51). All participants were informed regarding the aims and procedures of the study and they were reassured that the gathered information was kept confidential.

### Statistical analysis

Data analyses were performed by using the SPSS software (Statistical Package for the Social Sciences, version 16.0, SPSS Inc, Chicago, Illinois, USA). Then, we compared the characteristics of the two groups using two-tailed Student's *t*-test, Mann-Whitney U test, and Chi Square tests. The differences in the mean FSFI and MBSRQ scores between the two groups were assessed using Chi Square, Student's *t-*test, and Mann-Whitney U test. The relationship between the BMI, body image score, and FSFI were evaluated by the Pearson and Spearman correlation analysis tests. P-value < 0.05 was considered significant.

## 3. Results

A total of 206 women were included in this study: 106 normal weight (64 in the 2nd and 42 in the 3rd trimesters) and 100 overweight women (52 in the 2nd and 48 in the 3rd trimesters). The mean age of the pregnant women was 28.18 ± 5.66 yr (18-42), and the duration of marriage was 7.72 ± 5.26 yr. The mean of the pre-pregnancy BMI was 25.31 ± 3.97 (range 18.50-37.39), and the current BMI mean was 27.74 ± 4.20 (range 18.66-44.79). The mean number of pregnancies for each participant was 0.87 (range 0-4). Table I provides the general characteristics of study participants. There was no significant difference in the main characteristics of the two groups of normal and overweight women. The mean of the total FSFI score was 24.85 ± 5.15 (range 5.00-33.60). Of the 206 participants, approximately three quarters had female sexual disorder (FSD) (FSFI scores ≤ 28), with the prevalence of 72.3% (95% CI: 66.1%, 78.4%) (70.7% in 2nd and 74.4% in 3rd trimesters). The overall risk of sexual problem and all subscales were not significantly different among overweight and normal weight women, as can be seen in Table II. Diminished sexual desire/appetite was the most common problem reported by the participants (37.9%) compared to the other types of FSD. However, after dividing the participants into two groups, desire disorder in normal-weight women and arousal disorder in overweight women were the most common problem (Table II). The mean total score of FSFI were similar in the 2nd and 3rd trimesters (25.28 ± 4.52 vs. 24.29 ± 5.83; p = 0.170). For women in the 2nd and 3rd trimesters, mean scores of total FSFI and all the domains did not differ significantly between the overweight and normal weight women (Table III).

The relationship between the BMI and Body Image scores with FSFI were also evaluated. There were no relationships between BMI and Body Image with total FSFI and its subscales (Table IV). However, there was a significant relationship between the Appearance evaluation and Lubrication (p = 0.043). The relationships between subjective weight and two subscales of sexual satisfaction (p = 0.005) and orgasm (p = 0.019) were also significant (Table IV).

**Table 1:** The general characteristics of study participants


**Variable**	**Normal weight**	**Overweight**	**P-value**
Maternal age*	27.47 ± 5.53	28.94 ± 5.72	0.071
Gestational week*	26.25 ± 7.62	26.80 ± 7.57	0.607
Maternal body mass index*	22.19 ± 1.93	28.62 ± 2.70	0.0001
Duration of marriage*	7.21 ± 5.08	8.25 ± 5.44	0.144
Education**			
Less than diploma	28 (26.4)	22 (22.0)	
Diploma	37 (34.9)	39 (39.0)	
University education	41 (38.7)	39 (39.0)	<brow>-3</erow> 0.723
Husband education**			
Less than diploma	34 (32.1)	34 (34.0)	
Diploma	36 (34.0)	27 (27.0)	
University education	36 (34.0)	39 (39.0)	<brow>-3</erow> 0.540
Employment**			
Employed	18 (17.0)	9 (9.0)	
Housewife/unemployed	88 (83.0)	91 (91.0)	<brow>-2</erow> 0.090
Husband employment**			
Worker	27 (25.5)	28 (28.0)	
Employed	31 (29.2)	23 (23.0)	
Self-employed	48 (45.3)	49 (49.0)	<brow>-3</erow> 0.595
Planned pregnancy**			
No	22 (20.8)	28 (28.0)	
Yes	84 (79.2)	72 (72.0)	<brow>-2</erow> 0.147
Parity**			
0	45 (42.5)	34 (34.0)	
1	41 (38.7)	35 (35.0)	
2 and more	20 (18.9)	31 (31.0)	<brow>-3</erow> 0.122
*Data presented as mean ± SD; Mann-Whitney U test; **Data presented as n (%) Chi Square test			

**Table 2:** Sexual function in overweight women compared to the normal weight


	**Total**	**Normal weight**	**Overweight**	**P-value**
Female sexual function				
Yes	149 (72.3)	78 (73.6)	71 (71.0)	
No	57 (27.7)	28 (26.4)	29 (29.0)	<brow>-2</erow> 0.679
Desire disorder	78 (37.9)		
Yes	78 (37.9)	45 (42.5)	33 (33.0)	
No	128 (62.1)	61 (57.5)	67 (67.0)	<brow>-3</erow> 0.162
Pain disorder				
Yes	62 (30.1)	37 (34.9)	25 (25.0)	
No	144 (69.9)	69 (65.1)	75 (75.0)	<brow>-2</erow> 0.121
Lubrication disorder				
Yes	34 (16.5)	22 (20.8)	12 (12.0)	
No	172 (83.5)	84 (79.2)	88 (88.0)	<brow>-2</erow> 0.091
Satisfaction disorder				
Yes	28 (13.6)	17 (16.0)	11 (11.0)	
No	178 (86.4)	89 (84.0)	89 (89.0)	<brow>-2</erow> 0.292
Orgasm disorder				
Yes	37 (18.0)	23 (21.7)	14 (14.0)	
No	169 (82.0)	83 (78.3)	86 (86.0)	<brow>-2</erow> 0.150
Arousal disorder				
Yes	77 (37.4)	36 (34.0)	41 (41.0)	
No	129 (62.6)	70 (66.0)	59 (59.0)	<brow>-2</erow> 0.297
Data presented as n (%)				
Mann-Whitney U test and Chi Square tests				

**Table 3:** Female sexual function and body image scores of participants according to BMI in the 2nd and 3rd trimesters


**Variables**	**2nd trimester**			**3rd trimester**		
	**Normal weight**	**Overweight**	**P-value**	**Normal weight**	**Overweight**	**P-value**
Female sexual function	25.31 ± 4.61	25.25 ± 4.45	0.945	23.41 ± 6.61	25.06 ± 5.00	0.310
Desire	3.45 ± 0.93	3.42 ± 0.80	0.940	3.35 ± 0.89	3.48 ± 0.60	0.176
Pain	4.30 ± 1.26	4.32 ± 1.16	0.941	3.94 ± 1.31	4.21 ± 1.33	0.199
Lubrication	4.48 ± 1.10	4.65 ± 1.12	0.267	4.15 ± 1.76	4.40 ± 1.20	0.958
Satisfaction	4.88 ± 0.95	4.83 ± 0.99	0.941	4.65 ± 1.08	4.90 ± 0.84	0.336
Orgasm	4.42 ± 1.14	4.41 ± 1.06	0.882	3.84 ± 1.72	4.40 ± 1.28	0.117
Arousal	3.76 ± 0.96	3.60 ± 1.00	0.359	3.45 ± 1.47	3.64 ± 1.16	0.487
Data presented as mean ± SD; Mann-Whitney U test						

**Table 4:** Correlation between BMI, body image score and its subscales with female sexual function and its subscales


**Variables**	<**Female sexual function**		**Desire**		**Pain**		**Lubrication**		**Satisfaction**		**Orgasm**		**Arousal**	
	**r**	**p**	**r**	**p**	**r**	**p**	**r**	**p**	**r**	**p**	**r**	**p**	**r**	**p**
Body mass index	0.054	0.44	0.064	0.364	0.018	0.802	0.048	0.496	0.097	0.165	0.115	0.100	-0.028	0.687
Body image	-0.014	0.837	-0.015	0.833	-0.071	0.307	-0.015	0.832	0.001	0.996	0.019	0.781	-0.048	0.495
Appearance evaluation	-0.044	0.533	0.006	0.936	-0.106	0.128	-0.141${}^{*}$	0.041	-0.081	0.246	-0.134	0.054	-0.078	0.267
Fitness orientation	-0.051	0.468	-0.030	0.673	-0.025	0.719	-0.033	0.642	0.017	0.813	-0.091	0.194	-0.087	0.214
Fitness evaluation	-0.053	0.450	-0.100	0.151	-0.089	0.202	-0.074	0.293	-0.007	0.916	0.056	0.425	-0.027	0.696
Subjective weight	0.130	0.062	0.074	0.294	0.059	0.401	0.055	0.432	0.196${}^{**}$	0.005	0.163${}^{*}$	0.019	0.065	0354
Body areas satisfaction	0.104	0.136	0.073	0.294	0.028	0.694	0.122	0.082	0.047	0.507	0.091	0.195	0.036	0.607
Appearance orientation	-0.077	0.269	-0.039	0.577	-0.095	0.173	-0.096	0.171	-0.078	0.268	-0.037	0.594	-0.008	0.907
Statistical analysis was carried out using Pearson and Spearman correlation analysis tests														
*p < 0.05; **p < 0.005														

## 4. Discussion

The present study revealed that the prevalence of sexual problem is high during pregnancy. This finding was in agreement with a study by Jamali, indicating that the prevalence of FSD was 79.1% among Iranian pregnant women (4). The physiological and psychological changes in pregnancy may affect the sexual function in pregnant women. Indeed, couples' concerns about harming the baby during sexual intercourse may lead to misconception about sex and disruption in sexual function (22).

Further, the prevalence observed in this study was higher than that reported in the Brazilian pregnant adults in two studies (17, 23). In addition, a decrease or loss of desire/appetite was the most commonly reported sexual problem among the normal weight women, while the arousal problem was the most common sexual problem in overweight women. Jamali and Bayrami also reported the decrease of desire as the most common sexual problem among Iranian pregnant women (4, 24).

Additionally, a previous study also found that low desire was a more common disorder in women with longer partnerships (25). The diversity in sociodemographic and cultural characteristics, methods of assessment, and the difference in cut-off points used for determining FSD may explain some of the different findings regarding the prevalence rates between the previous and present studies. However, given that the primary aim of this study was not to estimate the prevalence of sexual functions, more studies in this population are required to confirm these findings.

In the present study, we found no significant association between the BMI score and FSFI in the 2nd and 3rd trimesters of pregnancy. This finding is consistent with the relevant findings from the two previous studies reporting no association between the FSFI score and BMI among Turkish and Brazilian pregnant women (26, 27). In contrast, a study by Ribeiro and colleagues claimed that overweight women had a worse sexual function in the 3rd but not in the 2nd trimester of pregnancy compared to the normal weight women (17). In another study, this author also suggested obesity as a risk factor for poor sexual function among women with Gestational Diabetes Mellitus (GDM) in the 3rd trimester of pregnancy (1). The different studies clarified the role of cultural prescriptions in shaping the response of women to sexual intercourse during pregnancy (28).

Therefore, these different results can be explained by the different sexual scripts that are dominant in the participants' culture (e.g., believing that a fat body is good enough to feed the baby and protect the pregnancy). Generally, there are only scarce data on the relationship between BMI score and the sexual functions of pregnant women. However, in the recent literature, some evidence have been published in non-pregnancy. For instance, these results are consistent with two studies that observed a no association between BMI and female sexual problem in non-pregnant women (29, 30).

Nevertheless, this is inconsistent with the findings of Esposito and co-workers, who found significant negative correlation between the BMI and the sexual function score in women with FSD but not those without FSD. Therefore, these authors claim that the effect of excess weight may become apparent after the development of sexual problem (31). As mentioned previously, the difference in scoring system as well as inclusion criteria, sample size, and diverse study backgrounds and attitude, and cultural differences among the different studies could explain the different findings on this subject.

In the present study, there was no relationship between body image score and sexual function. This finding was in agreement with Paul's results indicating that body image had no relationship with FSD during the postpartum period (32). Andersen also concluded that body image was not a predictor of female sexual function (33). However, this observation was inconsistent with the findings of most studies advocating the relationship between body image and sexual function in non-pregnant women (34, 35). One of the reasons for this discrepant finding seems to be that most women in this study reported a high score on the body's imagination. In this regard, one study found that body shape changes were better perceived and accepted by pregnant women (13).

Since, unlike Western studies, all the participants in this study were in a marital relationship, the perception of body may not change over the course of pregnancy. Thus, the relationship between body image and sexual function may be better interpreted by considering the partnered status in diverse populations (36). In support of this hypothesis, Pujols state that women who are in a partnered relationship are less concerned about their bodies during sexual activity and feel more comfortable with their partners over time (37).

Generally, the findings suggest that the sexual function of pregnant women may be driven by other factors such as physiological and psychological changes, other than BMI and body image.

The variables such as partners' reactions to the pregnancy, cultural expectations, ethical issues (e.g., sense of guilt engaging in sexual activity during pregnancy), misinformation and misconceptions regarding the harms of sexual intimacy in pregnancy (for instance, orgasm can cause contractions and lead to preterm birth), and lack of knowledge and skill about how to make sexual relation during pregnancy may better explain the high incidence of sexual problem in pregnancy in our population (2, 10, 11, 38-40). In this way, according to Uwapusitanon's study, the reduction of sexual activity during pregnancy was most often attributed to the beliefs of woman or partner about the possible risks of intercourse for the fetus (38). Furthermore, some studies suggested that some gynecologists and midwives prohibit pregnant women from intercourse without any medical indication and only due to the fear of abortion or preterm labor (39).

The present study, however, did have some limitations. First, due to the correlational design of this study, no causal relationship between studied variable can be claimed. Further, the assessment of sexual function by a self-report tool and non-objective evaluation can be other limitations of this study. In addition, the studied population is a sample of low-risk healthy Iranian pregnant women. Therefore, the generalizability of the findings to other population needs to be examined in future studies.

Regardless of this limitation, the findings of this study can provide valid information regarding the relationship between pre-pregnancy BMI, body image score, and sexual function in pregnancy. However, due to the lack of correlation between the studied variables, it is useful to investigate other aspects that could have affected the sexual function in pregnancy. Furthermore, future research needs to assess these findings in other cultures or socioeconomic strata with more in-depth investigations.

## 5. Conclusion

In conclusion, the current study revealed that there were no relationships between the BMI plus body image score and sexual function. Therefore, based on the results of this study, intricate constructs such as sexual function cannot be explained by the concepts such as BMI and body image. Accordingly, further research is recommended to study other potential factors affecting sexual function during pregnancy. However, given the high prevalence of sexual problems in this population during pregnancy, healthcare providers should investigate the sexual function of the expectant mothers and be able to recommend possible solutions.

##  Conflict of Interest

The authors have no conflicts of interest to declare.

## References

[1]   Ribeiro MC, Nakamura MU, Torloni MR, Scanavino Mde T, Scomparini FB, Mattar R. Female sexual function of overweight women with gestational diabetes mellitus - a cross-sectional study. *PloS One* 2014; 9: e95094..

[2]   Trutnovsky G, Haas J, Lang U, Petru E. Women's perception of sexuality during pregnancy and after birth. *Aust N Z J Obstet Gynaecol* 2006; 46: 282–287..

[3]   Seven M, Akyuz A, Gungor S. Predictors of sexual function during pregnancy. *J Obstet Gynaecol *2015; 35: 691–695..

[4]   Jamali S, Mosalanejad L. Sexual dysfnction in Iranian pregnant women. *Iran J Reprod Med* 2013; 11: 479–486..

[5]   Yeniel AO, Petri E. Pregnancy, childbirth, and sexual function: perceptions and facts. *Int Urogynecol J *2014; 25: 5–14..

[6]   Williamson M, McVeigh C, Baafi M. An australian perspective of fatherhood and sexuality. *Midwifery* 2008; 24: 99–107..

[7]   Bartellas E, Crane JM, Daley M, Bennett KA, Hutchens D. Sexuality and sexual activity in pregnancy. *BJOG* 2000; 107: 964–968..

[8]   Mozafari M, Khajavikhan J, Jaafarpour M, Khani A, Direkvand-Moghadam A, Najafi F. Association of body weight and female sexual dysfunction: a case control study. *Iran Red Crescent Med J* 2015; 17: e24685..

[9]   Ramezani Tehrani F, Farahmand M, Simbar M, Malek Afzali H. Factors associated with sexual dysfunction; a population based study in Iranian reproductive age women. *Arch Iran Med* 2014; 17: 679–684..

[10]   Weaver AD, Byers ES. The relationships among body image, body mass index, exercise, and sexual functioning in heterosexual women. *Psychol Women Quarter *2006; 30: 333–339..

[11]   Afshari P, Houshyar Z, Javadifar N, Pourmotahari F, Jorfi M. The relationship between body image and sexual function in middle-aged women. *Electron Physician* 2016; 8: 3302–3308..

[12]   Widen EM, Gallagher D. Body composition changes in pregnancy: measurement, predictors and outcomes. *Eur J Clin Nutr* 2014; 68: 643–652..

[13]   Fuller-Tyszkiewicz M, Skouteris H, Watson BE, Hill B. Body dissatisfaction during pregnancy: a systematic review of cross-sectional and prospective correlates. *J Health Psychol* 2013; 18: 1411–1421..

[14]   Manaf NA, Saravanan C, Zuhrah B. The prevalence and inter-relationship of negative body image perception, depression and susceptibility to eating disorders among female medical undergraduate students. *J Clin Diagn Res* 2016; 10: VC01–VC04..

[15]   Rahmani A, Sayehmiri K, Asadollahi K, Sarokhani D, Islami F, Sarokhani M. Investigation of the prevalence of obesity in iran: a systematic review and meta-analysis study. *Acta Med Iran* 2015; 53: 596–607..

[16]   Felisbino-Mendes MS, Matozinhos FP, Miranda JJ, Villamor E, Velasquez-Melendez G. Maternal obesity and fetal deaths: results from the Brazilian cross-sectional Demographic Health Survey, 2006. *BMC Pregnancy Childbirth* 2014; 14: 5–15..

[17]   Ribeiro MC, Nakamura MU, Torloni MR, Scanavino Mde T, Mancini PE, Forte BM, *et al*. Maternal overweight and sexual function in pregnancy. *Acta Obstet Gynecol Scand* 2016; 95: 45–51..

[18]   Rosen R, Brown C, Heiman J, Leiblum S, Meston C, Shabsigh R, *et al*. The female sexual function index (fsfi): a multidimensional self-report instrument for the assessment of female sexual function. *J Sex Marit Ther* 2000; 26: 191–208..

[19]   Mohammadi Kh, Heydari M, Faghihzadeh S. [The female sexual function index (FSFI): validation of the Iranian version]. *Payesh* 2008; 7: 269–278. (in Persian).

[20]   Cash TF. The body image work book (An 8-step program for learning to like your looks). Oakland: New Harbinger Publications; 1997..

[21]   Rahati A. Evolutionary study of body image and its relationship with self-esteem based on comparison between adolescent, middle age and old people. Tehran: Shahed University; 2004..

[22]   Shojaa M, Jouybari L, Sanagoo A. The sexual activity during pregnancy among a group of Iranian women. *Arch Gynecol Obstet* 2009; 279: 353–356..

[23]   Leite AP, Campos AA, Dias AR, Amed AM, De Souza E, Camano L. Prevalence of sexual dysfunction during pregnancy. *Rev Assoc Med Bras* 2009; 55: 563–568..

[24]   Bayrami R, Sattarzadeh N, Ranjbar Koochaksariie F, Zakaria Pezeshki M. [Sexual dysfunction in couples and its related factors during pregnancy.] *J Reprod Infertil* 2008; 9: 271–283. (in Persian).

[25]   Hayes RD, Dennerstein L, Bennett CM, Sidat M, Gurrin LC, Fairley CK. Risk factors for female sexual dysfunction in the general population: exploring factors associated with low sexual function and sexual distress. *J Sex Med* 2008; 5: 1681–1693..

[26]   Tasdemir N, Celik C, Abali R, Ozer I, Gokhan Culha M, Serefoglu EC. Sexual function may be impaired during pregnancy in adolescent women. *Sexual and Relationship Therapy* 2017; 32: 173–180..

[27]   Naldoni LM, Pazmino MA, Pezzan PA, Pereira SB, Duarte G, Ferreira CH. Evaluation of sexual function in Brazilian pregnant women. *J Sex Marital Ther* 2011; 37: 116–129..

[28]   Sossah L. Sexual behavior during pregnancy: A descriptive correlational study among pregnant women. *Eur J Res Med Sci *2014; 2: 16–27..

[29]   Faridi H, Najar Sh, Javadnoori M. The relationship between body mass index and women sexual function. *Iran J Obstet Gynaecol Infertil* 2013; 16: 20–28..

[30]   Abidin A, Draman N, Ismail S, Mustaffa I, Ahmad I. Female sexual dysfunction among overweight and obese women in Kota Bharu, Malaysia. *J Taibah Uni Med Sci* 2016; 11: 159–167..

[31]   Esposito K, Ciotola M, Giugliano F, Bisogni C, Schisano B, Autorino R, *et al*. Association of body weight with sexual function in women. *Int J Impot Res* 2007; 19: 353–357..

[32]   Pauls RN, Occhino JA, Dryfhout VL. Effects of pregnancy on female sexual function and body image: a prospective study. *J Sex Med* 2008; 5: 1915–1922..

[33]   Andersen BL, Legrand J. Body image for women: conceptualization, assessment, and a test of its importance to sexual dysfunction and medical illness. *J Sex Res* 1991; 28: 457–477..

[34]   van den Brink F, Smeets MA, Hessen DJ, Woertman L. Positive body image and sexual functioning in dutch female university students: the role of adult romantic attachment. *Arch Sex Behav* 2016; 45: 1217–1226..

[35]   Woertman L, van den Brink F. Body image and female sexual functioning and behavior: a review. *J Sex Res* 2012; 49: 184–211..

[36]   Satinsky S, Reece M, Dennis B, Sanders S, Bardzell S. An assessment of body appreciation and its relationship to sexual function in women. *Body Image* 2012; 9: 137–144..

[37]   Pujols Y, Seal BN, Meston CM. The association between sexual satisfaction and body image in women. *J Sex Med* 2010; 7: 905–916..

[38]   Uwapusitanon W, Choobun T. Sexuality and sexual activity in pregnancy. *J Med Assoc Thai *2004; 87 (Suppl.): 45–49..

[39]   Heidari M, Amin Shokravi F, Zayeri F, Azin SA, Merghati-Khoei E. Sexual life during pregnancy: effect of an educational intervention on the sexuality of iranian couples: a quasiexperimental study. *J Sex Marital Ther* 2018; 44: 45–55..

[40]   Afshar M, Mohammad-Alizadeh-Charandabi S, Merghti-Khoei ES, Yavarikia P. The effect of sex education on the sexual function of women in the first half of pregnancy: a randomized controlled trial. *J Caring Sci* 2012; 1: 173–181..

